# Non-financial access to healthcare services in rural areas: A case study of people with disabilities living in Northern Iran

**DOI:** 10.1371/journal.pone.0289583

**Published:** 2023-12-12

**Authors:** Lida Shams, Tahere Darvish, Mohamad Meskarpour Amiri, Sayyed-Morteza Hosseini-Shokouh, Taha Nasiri

**Affiliations:** 1 Department of Health Management, Policy and Economic, Virtual School of Medical and Management, Shahid Beheshti University of Medical Sciences, Tehran, Iran; 2 Department of Community Health Education, Virtual School of Medical and Management, Shahid Beheshti University of Medical Sciences, Tehran, Iran; 3 Health Management Research Center, Baqiyatallah University of Medical Sciences, Tehran, Iran; 4 Department Of Health Services Management, Faculty Of Health, Baqiyatallah University Of Medical Sciences, Tehran, Iran; Shahid Beheshti University of Medical Sciences School of Dentistry, ISLAMIC REPUBLIC OF IRAN

## Abstract

**Introduction:**

Access to healthcare for persons with disabilities (PWDs) is an important but often ignored issue for achieving universal health coverage. The current study aimed to investigate PWDs’ access to healthcare in the rural areas in north of Iran.

**Methods:**

Following a descriptive-analytical design, 471 persons with disabilities (PWDs) living in the Nor city, Mazandaran province, were selected using quota sampling. Data were collected by a valid and reliable questionnaire that contained dimensions of time, geography, physical, and acceptability using face-to-face interviews. The findings are provided by central and dispersion indicators and analyses are performed with linear Regression using SPSS version 17.

**Results:**

PWDs had moderate access to healthcare services in all dimensions. The regression models for access to health services in all four dimensions were significant (p<0.05). The results showed that in the geographical dimension, the variables of marital status, income, receipt of financial aid, supplementary insurance, and type of disability; in the physical dimension, the variables of income, responsibility for taking care of the family, supplementary insurance, and type of disability; in the time dimension, supplementary insurance, home area, and type of disability; and in the aspect of service acceptability, only the variables of type of disability and internet access had a significant effect (p<0.05).

**Conclusion:**

A small percentage of PWDs had high access to health services. Hence, improving their access to healthcare services, particularly in rural and less developed areas, and developing appropriate policies should be the focus of Iranian policy-makers.

## Introduction

Promoting health equity for all people in society, regardless of their economic and social differences, is a major goal of policymakers [1]. As one of the main components of universal health coverage (UHC), access is a valuable tool to achieve health equity [2, 3]. Society only uses services that are acceptable and affordable [4]. Access is the power and ability to take actions in order to use medical resources in any circumstances and means providing appropriate services at the right time and in the right place [5]. There are various types of access, including physical, technological, economic, and time [6].

Research results in the U.S. indicated that improved provision and access to healthcare services resulted in reduced overall mortality by 22/9%. In addition, the evidence indicated that improved medical conditions caused a 5-year increase, on average, in life expectancy [5]. One study in Iran mentioned the undesirable status of access to healthcare services in various regions of Iran, i.e., some services are needed and not obtained [7].

Achieving UHC is a key goal for the World Health Organization (WHO) [8]. About 650 million PWDs and their family members, around 2 billion, are directly affected by disabilities, and providing healthcare services to them is an important but often ignored challenge [9]. PWDs make up 15% of the global population according to the International Labour Organization [10]. Similar to the global rate, in 2006, about 10% of Iranians were suffering from a type of disability, i.e., physical, cognitive, mental, or social [11]. PWDs are more likely to develop various chronic diseases and the prevalence of diseases such as asthma, cardiovascular disease, and hypertension is higher among them [12]. Hence, they tend to have a higher need for healthcare services. Tomlinson et al. (2007). reported that even in rich countries access to healthcare services is often difficult for PWDs and is more severe in low-income countries [12]. Unaffordability and high transportation costs are two main reasons that PWDs do not receive the necessary care in low-income countries [4].

As the first step to providing an appropriate response to the needs of PWDs is the identification of their problems. The current study aimed to investigate their access to healthcare services, in terms of time, geographical, and physical access as well as acceptability, in a sample of PWDs living in rural areas in the north of Iran. Acceptability means access acceptability Health care providers respond to the social and cultural expectations of their communities. Insurance coverage, income, health care costs, information, transportation, and communications play a key role in PWDs’ access to health services [13]. Also, seeking and analyzing factors associated with poor access intend to help policymakers in identifying and addressing barriers to access health services.

## Methods

### Design

This descriptive, analytical, applied study was conducted cross-sectionally in 14 comprehensive rural healthcare centers (CRHC) in Noor City of Mazandaran province in 2019.

### Participants and data collection procedure

A total of 62 466 people were living in the catchment areas of investigated CRHCs. The sample size was estimated as 471 subjects, based on the prevalence of 15% for disabilities in the study by [14] with a 95% confidence interval and a precision of 0/04.

In this study, the definition provided by the WHO for disability was used, which includes people who could not do their daily activities without the help of a caregiver during the past six months, including difficulty in standing for long periods (e.g., up to 30 minutes), family-related affairs, learning a new skill (e.g., how to go to a new location), participating in social activities (e.g. celebrations or religious activities), emotional effects of health status, difficulty in concentration for more than 10 minutes, difficulty in walking a long distance (e.g., one kilometer), difficulty in bathing and getting dressed, difficulty in communicating with people they don’t know, difficulty in maintaining a friendly relationship, and difficulty in doing daily activities [14]. Healthy cases, those younger than 16, and pregnant women were excluded.

Participants were recruited using cluster sampling. The catchment area of CRHCs contains a main village and a number of satellite villages. In this line, 47,257 cases were recruited from main villages and 15,209 from satellite villages. Participants were recruited proportionate to the total population of villagers using random selection sampling. Data were collected using face-to-face interviews and a researcher developed questionnaire that contained seven dimensions of healthcare service utilization, financial ability, service provision, geographical accessibility, physical accessibility, time accessibility, and acceptability.

### Data analysis

This study is part of a larger research that intended to investigate seven dimensions of access including availability, financial ability, and utilization of healthcare services, time access, geographical access, and physical access as well as acceptability. This study only examined non-financial aspects of access, including physical, time, geographical, and acceptability. As the applied questionnaire was designed for urban areas, its validity and reliability were evaluated. Content validity and face validity were evaluated by 8 experts. First, the experts were contacted in person or by phone, and after obtaining their permission and receiving their e-mail address or sending through automation, the content validity form of the questionnaire was sent and each question in the questionnaire was answered by three options: "relevant and important", "not necessary" and " "Irrelevant" was questioned and after receiving, the comments were entered into the Excel software environment and the validity of the content was calculated using Lawashe method. In this formula, ne is the number of necessary answers and n is the number of subjects.


CVR=ne‐n/2/n/2

Using the Lawshe test, 4 items with a content validity of < 0/75 were removed. In addition to the opinions of experts, the viewpoint of the study population was also used to evaluate face validity (comprehensibility and acceptance of the questionnaire).To assess the reliability, the questionnaire was piloted on a sample of 30 subjects by the test-retest method and all coefficients in all dimensions were higher than 72%. In order to measure the level of access to health services, all figures obtained in four dimensions, including geographic access in the form of a Likert scale (all, some, no), Physical access in the form of a Likert scale (most of the time, sometimes and no), Time access in the form of a Likert scale (most of the time, sometimes, and no), the Acceptability of services (on a Likert scale ranging from a lot, somewhat, and no, were combined, i.e., converted to an interval variable for each respondent (Table 1). And the higher the value, the better the access.

**Table 1:** Practical evaluation of the concept of access to healthcare services.

Level of access to healthcare services	Values
Very low access	less than 3 points
low access	(3–6) points
Average access	(6–9) points
High access	(9 and above) points

To determine the relationship between contextual factors (age, gender, marital status, education level, employment status, head of household income, head of household, receiving financial aid, basic insurance, supplementary insurance, home area, place of residence, type of disability, type of insurance, Having a car, having the Internet) regression was used. The findings are provided using central and dispersion indicators and analyses are performed with Linear Regression using SPSS version 17.

### Ethical consideration

A questionnaire was used in the method of work and considering that if the participants were not willing to participate in the study, the participants would definitely withdraw from the study. On the other hand, verbal consent should be obtained from the participants. Therefore, those who had informed consent have completed the questionnaire. Also, this study has been extracted from the master’s thesis of Shahid Beheshti University of Medical Sciences and has obtained the necessary license from the Research Ethics Committee of Shahid Beheshti University of Medical Sciences of Iran with the code IR.SBMU.SME.REC.1397.004.

## Results

Sixty percent of the participants were women and most of them were over 60 years old. In addition, in almost 90% of the participants, the monthly income of the head of the household was less than 20 million Rials. Also, 200 participants were married, 357 were illiterate, and mostly unemployed. Twenty percent of the participants were heads of households and 18 percent received financial aid. 54 percent of the disabled were over 60 years old. Physical disability was the most important form of disability. Ninety-three percent had basic insurance and seventy-eight percent needed supplementary insurance funds. In addition, 95 percent of them owned a home with an area of 50 to 100 m^2.^ The demographic characteristics of participants are provided in Table 2.

**Table 2:** Demographic and socioeconomic characteristics of the participants.

percentage	number	variable		percentage	number	variable	
6/36	93	Less than 10 million Rial	**Income of the head of the household**	40	188	Male	**Gender**
1/53	135	10 to 20		60	281	Female	
3/10	26	More than 20		3/11	53	16 tp 29 years (young)	**Age Group**
1/20	94	The disabled person	**Head of the household**	5/34	162	30 to 60 years (adult)	
1/26	122	Father		2/54	254	Older than 60 years (elder)	
15	70	Wife		6/42	200	Married	**Marital Status**
9/25	121	Child		3	14	Divorced	
13	60	Other (brother or mother)		3/31	147	Not married	
18	84	Yes	**Financial aid**	23	108	Other	
82	385	No		1/76	357	Illiterate	**Education Level**
1/5	24	From birth	**Age of disability**	1/19	90	Less than diploma	
2/6	29	Less than 30 years old		4/3	16	Diploma	
5/34	162	30 to 60 years		4/1	6	Higher than diploma	
2/54	254	Older than 60 years		1/1	5	Full-time	**Employment Status**
93	436	Yes	**Primary insurance coverage**	9/6	32	Part time	
7	33	No		5/92	432	No	
3/21	100	Yes	**Complimentary insurance coverage**	5/53	251	Physical	**Type of Disability**
7/78	369	No		5/27	129	Cognitive	
7/10	50	Less than 50 meter	**Area of residence**	17	80	Physical and cognitive	
9/68	323	50 to 100 m		7/94	444	Personal	**Residence status**
4/16	77	100 to 150 m		3	14	Rental	
1/4	19	150 to 200 m		3/2	11	Other	

The results showed that the mean score of accessibility of disabled people to health services in terms of “geographical access” was 7/83 ± 1/76 and in terms of 273 people (58/2%) of the study samples the level of access in this dimension was also average and only 16/6% of them have overestimated the level of geographical access. Also, the average score of access to health services in the dimension of “physical access” in the point of the disabled was 7/43± 1/70 and the level of access in this dimension in terms of most of the studied samples (335 people, equivalent to 71/4%) was in the average range. The mean score of access to health services in terms of “time access” was 8/87 ± 1/76 and in terms of 242 people (51/6%) of the samples, access to health services required in terms of time was average and 39/7% of the samples evaluated the level of access in this dimension as high. The results also showed that the mean score of access to health services in the service acceptability dimension was 23/75 ±2/93 and, in terms of 271 people (57/8%) of the samples, access to the required health services in terms of time was moderate and 39/7% of the samples rated the level of access in this dimension as high (Table 3).

**Table 3:** Average and frequency distribution of access to health services in geographical, physical, time dimensions and acceptability of services in terms of studied samples.

Access Dimension		Subgroup (score)	Number	%	Mean	±SD
**Dimensions of access to health services for people with disabilities**	Geographical access	Very low access(less than 3)	0	0	83/7	76/1
		Low access (3–6)	118	25.2		
		Average access (6–9)	273	58.2		
		High access (9 and above)	78	16.6		
	Physical access	Very low access (less than 3)	20	4.3	43/7	70/1
		Low access (3–6)	114	24.3		
		Average access (6–9)	335	71.4		
		High access (9 and above)	0	0		
	Time access	Very low access (less than 3)	0	0	87/8	76/1
		Low access (3–6)	41	8.7		
		Average access (6–9)	242	51.6		
		High access (9 and above)	186	39.7		
	Service acceptability	Very low access (less than 10)	0	0	75/23	93/2
		Low access (10–20)	65	13.9		
		Average access (20–30)	271	57.8		
		High access (30 and up)	133	28.4		

Also, the most important barriers for the disabled to access health services are included in the table below (Table 4).

**Table 4:** Barriers to access to healthcare services.

Dimension		Range	%	Dimension		Range	%
**Time**	**Waiting time in medical centers**	High	16/6	***Acceptability***	**Inappropriate quality of medical and rehabilitative services**	Mostly	**3/6**
		Moderate	51/8			Often	**31**
		Low	31/6			Never	**65//4**
	**Walking for a long time to reach health centers**	Mostly	12/8		**Disrespectful behavior**	Mostly	**4/4**
		Often	38			Often	**9**
		Never	49/2			Never	**86/6**
	**Delays in receiving services due to long waiting**	Mostly	12/2		**Negative judgment and attitude of employees**	Mostly	**1/3**
		Often	48/4			Often	**10/7**
		Never	39/4			Never	**88**
	**Incompatibility of response hours**	Mostly	35/2		**Language barriers (i.e., not speaking a the local accent)**	Mostly	**52/3**
		Often	38/8			Often	**43**
		Never	26			Never	**4/7**
**Physical**	**Housing problems (high number of stairs, lack of elevators or ramps)**	High	11		**Possibility to talk with the doctor and/or nurses in private**	Mostly	**22**
		Moderate	29/8			Often	**42**
		Low	59/2			Never	**36**
	**Inappropriate roads, sidewalks, and pedestrian bridges**	Mostly	10/5		**Not listening to PWDs**	Mostly	**43/2**
		Often	33/5			Often	**47/2**
		Never	56			Never	**9/6**
	**Non-compliance with the appropriateness of medical centers for PWDs (high stairs, lack of elevators or ramps)**	Mostly	9.3		**Providing health information**	Mostly	**59/7**
		Often	31			Often	**36/8**
		Never	59/7			Never	**3/5**
**Geographical**	**Long distance**	Mostly	**9**		**Family support**	High	**48.5**
		Often	**23**			Moderate	**42.4**
		Never	**58.8**			Low	**9.1**
	**Access to services near a living area**	Public health services	65.2		**Friends’ support**	High	**14**
		Some public health services	23.5				
		All medical services	8.7				
		Some medical services	22.3			To somehow	**34.3**
		All rehabilitative services	9			No	**51.7**
		Some rehabilitative services	22				

Along with other barriers; In the dimension of time access, 51/8% of the respondents evaluated the waiting time in the medical center to see a doctor or other personnel as average. In the dimension of service acceptability, 42/4% of the clients had the necessary support to some extent. Also, 42% sometimes have the possibility to talk with doctors and nurses privately. 47/2% of health workers sometimes take time to listen to the problems of disabled people. And 43% of health workers can sometimes speak to disabled people in the language they prefer.

The Results showed that in general, regression models of access to health services in all four dimensions of geographical (F = 3/218 and P = 0/001), physical (F = 2/599 and P = 0/001), time (F = 3/550 and P = 0/001) and acceptability (F = 3/711 and P = 0/001) are significant. On the other hand, some variables including marital status, household income, receiving financial aid, supplementary insurance and type of disability had significant relationship with geographical access. At the physical access dimension the effect of variables including household income, head of household, supplementary insurance and type of disability was significant. Also in the time dimension variables including education level, basic and supplementary insurance, home area and type of disability had significant relationship with time access. In the dimension of service acceptability the effect of disability type and access to internet were significant (P <0/05) (Table 5).

**Table 5:** Results of regression estimates between study variables and access to health services.

Acceptance of services			Time			Geographical			Physical			Variables	
p-value	Statistics t	Beta	p	Statistics t	Beta	p-value	Statistics t	Beta	p-value	Statistics t	Beta		
**399/0**	**845/0-**	**317/0-**	**210/0**	**257/1-**	**280/0-**	**401/0**	**842/0**	**195/0**	**100/0**	**651/1-**	**426/0-**	**Gender**	**Independent variables**
**319/0**	**998/0**	**154/0**	**239/0**	**182/1**	**108/0**	**506/0**	**667/0-**	**064/0-**	**040/0**	**071/2**	**220/0**	**Marital status**	
**145/0**	**461/1-**	**468/0-**	**040/0**	**068/2-**	**393/0-**	**192/0**	**310/1-**	**260/0-**	**300/0**	**038/1-**	**229/0-**	**Level of education**	
**283/0**	**077/1**	**758/0**	**303/0**	**032/1-**	**432/0-**	**538/0**	**617/0-**	**269/0**	**777/0**	**283/0**	**137/0**	**Job status**	
**118/0**	**570/1-**	^**7–**^ **436/4-**	**095/0**	**678/1-**	^**7–**^ **816/2-**	**015/0**	**462/2-**	^**7–**^ **310/4-**	**023/0**	**283/2-**	^**7–**^ **444/4-**	**household head income**	
**155/0**	**426/1-**	**301/0-**	**413/0**	**820/0-**	**103/0-**	**023/0**	**291/2-**	**299/0-**	**936/0**	**080/0**	**012/0**	**head of household**	
**515/0**	**653/0**	**368/0**	**638/0**	**471/0-**	**158/0-**	**922/0**	**098/0**	**034/0**	**001/0**	**910/3**	**519/1**	**Receiving financial aid**	
**640/0**	**468/0-**	**331/0-**	**045/0**	**018/2**	**847/0**	**773/0**	**288/0**	**126/0**	**165/0**	**392/1-**	**678/0-**	**Basic insurance**	
**328/0**	**980/0**	**430/0**	**043/0**	**033/2**	**530/0**	**005/0**	**833/2**	**770/0**	**002/0**	**068/3**	**927/0**	**Supplementary insurance**	
**831/0**	**213/0**	**002/0**	**021/0**	**334/2-**	**011/0**	**426/0**	**798/0**	**004/0**	**010/0**	**600/2**	**014/0**	**Home area**	
**807/0**	**244/0-**	**003/0-**	**913/0-**	**109/0-**	**001/0-**	**053/0**	**945/1**	**016/0**	**033/0**	**150/2**	**019/0**	**Age**	
**166/0**	**392/1-**	**373/0-**	**013/0**	**512/2-**	**399/0-**	**950/0**	**063/0**	**010/0**	**837/0**	**206/0-**	**038/-**	**place of residence**	
**001/0**	**399/3-**	**745/0-**	**001/0**	**291/3**	**429/0**	**011/0**	**575/2**	**350/0**	**889/0**	**140/0**	**021/0**	**Type of disability**	
**696/0**	**391/0-**	**039/0-**	**693/0**	**396/0-**	**024/0-**	**909/0**	**114/0-**	**007/0-**	**693/0**	**396/0-**	**027/0-**	**Type of insurance**	
**684/0**	**408/0**	**167/0**	**743/0**	**328/0**	**080/0**	**118/0**	**570/1-**	**398/0-**	**438/0**	**777/0-**	**219/0-**	**Car ownership**	
**001/0**	**264/4-**	**874/1-**	**239/0**	**180/1**	**308/0**	**828/0**	**217/0-**	**059/0-**	**054/0**	**936/1**	**586/0**	**Access to internet**	
**001/0**			**001/0**			**001/0**			**001/0**			**p-value**	**index of fit**
**221/0**			**214/0**			**166/0**			**198/0**			**R Square**	
**711/3**			**550/3**			**599/2**			**218/3**			**F**	
**711/1**			**485/1**			**450/1**			**835/1**			**Durbin-Watson**	

## Discussion

The study results showed that the accessibility of disabled people to health services in terms of geographical access, physical access, time access, and service acceptability was at a moderate level. Therefore, our results revealed moderate access of PWDs to healthcare services in all four dimensions. In a similar study by Wongkongdech et al. (2014) in Thailand, 66% of people with physical disabilities of all ages rated their access to health services as moderate (i.e., neither high nor low) [14] In another study by Vergunst et al., access to health care for people with disabilities in rural South Africa, 24.4% of disabled people reported that they needed health services and did not receive them, while this percentage was 12.6% for non-disabled people. It has also been shown that people with disabilities (PWDs) are more likely to have poorer general health [15–17]. The results of our research and review of existing literature revealed that in developing countries, necessary measures should be taken to improve PWDs’ access to healthcare services in all four dimensions, including geographical access, physical access, time access, as well as service acceptability.

The study results indicate that the most common access barriers reported by PWDs were long walking distances to reach health centers, extended waiting times in health centers, inconvenient response hours, inadequate opportunities to communicate privately with healthcare providers, and inadequate listening to PWDs and provision of health information. Health policymakers should pay greater attention to these barriers to improve the accessibility of the disabled. Similar barriers have been reported in related studies in other developing countries. For instance, in Morocco, 25% of people faced the problem of traveling long distances to access healthcare services [13]. In a study in Iran, veterans identified long waiting times for medical appointments or consultations as their most significant issue [18]. Another study in Iran examined the status of access to health and treatment centers for PWDs in Shiraz and found that 52 percent of disabled people believed that different parts of health centers, including elevators, corridors and passageways, patient waiting rooms and furniture, public office areas, ramps, restrooms, doors and windows, stairs, the main entrance of the building, and the parking lot, were not suitable for disabled people [19].

According to the results of our study, a significant proportion of PWDs reported dissatisfaction with the language and dialect used by healthcare personnel, and also the lack of opportunity for private communication. A meta-synthesis study by Hashemi et al. identified the absence of translators for those with hearing impairments as the primary barrier to accessing healthcare services [19]. Other studies have reported that PWDs in developing countries often have cultural and social concerns about the healthcare services they receive [20, 21]. A study conducted in South Africa found that only 4.4% of PWDs were dissatisfied with the behavior of healthcare staff [20]. PWDs expect their rights to be respected when accessing healthcare services. Reiss et al. demonstrated that good communication skills among healthcare staff were crucial for ensuring access to healthcare services and should be focused on patient-centered communication [22]. Therefore, policymakers should prioritize plans to improve the knowledge of healthcare staff on the rights, social, and cultural needs of PWDs.

The results of the regression models showed that there was a significant relationship between access to health services and all four dimensions of geographical (F = 3.218 and P = 0.001), physical (F = 2.599 and P = 0.001), time (F = 3.550 and P = 0.001), and acceptability (F = 3.711 and P = 0.001). Furthermore, the socio-economic status of PWDs was significantly related to their access to healthcare services in all four dimensions of geographical, physical, time, and acceptability. These factors included marital status, household income, insurance status, and the education level of PWDs. Therefore, it is necessary to address socio-economic inequalities among PWDs to improve their accessibility and health status. The presence of such inequalities may lead to the development of inequality in access to healthcare services in all four dimensions. Despite the increasing tendency in recent years to measure and document health inequalities and their socio-economic determinants for the purpose of health policies, studies in this regard are scarce among PWDs. For example, Raisi Dana et al. reported the lack of access to counseling and supportive services by families with PWD as a weakness in the health system in Iran [23], while the WHO also emphasized the need for more equitable access to these services [24]. In South Africa, a study showed that PWDs have higher levels of unmet needs compared to those without disabilities, mainly due to barriers in accessing healthcare services [25, 26].

## Conclusion

Improving adequate access to healthcare, in line with universal health coverage, is a crucial goal that can reduce the effects of poverty and inequalities. However, it is also important to recognize and address the barriers that PWDs face in accessing healthcare services, as they are a potential indicator of the success of the country’s health system efforts in improving access and utilization, especially for PWDs and the poor. Policy makers can support community health programs or community-directed interventions that bring health services closer to the people, such as training and deploying community health workers, volunteers or traditional healers to provide basic health care, referrals and follow-ups for PWDs in their homes or villages. Policy makers can also expand outreach services or mobile clinics that provide periodic visits of specialized health professionals or teams to rural areas to offer health services that are otherwise unavailable or inaccessible for PWDs. Moreover, policy makers can leverage telemedicine to improve access to health services for PWDs in rural areas by using information and communication technologies, such as phone, internet, video conferencing, etc., to facilitate remote consultation, diagnosis, treatment, education and monitoring of PWDs.

## Supporting information

S1 Data (SPV)

S2 Data (SPV)

S3 Data (SPV)

S4 Data (SPV)

S1 Appendix (DOCX)
