# Supplementary material for: Non-financial access to healthcare services in rural areas: A case study of people with disabilities living in Northern Iran
Source: PLoS One. 2023 Dec 12;18(12):e0289583. doi: 10.1371/journal.pone.0289583 (PMC10715662; doi:10.1371/journal.pone.0289583)
Supplement: S1 Appendix — (DOCX) [file pone.0289583.s005.docx]

**Appendix**

Noor city has three central parts, Beldeh and Chamestan, and its important villages are Abbasa, Tashkoh, Lavij, and Seliakti. Some villages of this city are far from Noor city, such as Shir Kala, which is 33 kilometers away from Noor city. Considering that the rural population of Noor city is 62466 people, then using the random selection table in the order of 1 Banafsheh Deh center 2 (with 12 main villages and 13 moons) - Seliakti center (4 main villages and 6 moons) 3- Aho Dasht center (5 main villages and 3 moons) 4- Lavij center (4 main villages and 5 moons) were selected as clusters. Those interviews with disabled people are conducted in the main and Qamar villages of these centers. The number of interviewees in each village is distributed according to the population of each village. In order to select the samples in Qamar and Main villages, according to the population of the main villages (47257 people) and Qamar village (15209 people), the number of samples will be proportionally distributed between them, so it is necessary to select 356 disabled people in the main villages and 115 disabled people. Interviews should be conducted in Qamar villages

According to calculations in Banafsheh Deh Comprehensive Health Service Center (93 people in the main villages and 67 people in Qamar villages), in Seliakti Comprehensive Health Service Center (102 people in the main villages and 23 people in Qamar villages), in Aho Dasht Comprehensive Health Service Center (108 people in the villages Main and 11 people in Qamar villages) and in Lavij comprehensive health service center (53 people in main villages and 14 people in Qamar villages) were interviewed face to face.
